# Designing for impact: Introducing translational design studies

**DOI:** 10.1017/cts.2026.10777

**Published:** 2026-07-10

**Authors:** Boris Volkov, Reuben Adatorwovor, Lloyd Michener, Christopher Lindsell, Justin B. Moore

**Affiliations:** 1 CTSI, University of Minnesota, Minneapolis, MN, USA; 2 Institute for Informatics, Data Science, and Biostatistics, Washington University in St Louis, St. Louis, MO, USA; 3 Department of Emergency Medicine, School of Medicine, Washington University in St Louis, St. Louis, MO, USA; 4 Community and Family Medicine and Duke University Clinical and Translational Science Institute, Durham, NC, USA; 5 Duke Clinical Research Institute and Duke Clinical and Translational Science Institute, Duke University, Durham, NC, USA; 6 Department of Implementation Science, Wake Forest University School of Medicine, Winston-Salem, NC, USA

**Keywords:** Clinical and translational science, translational design, formative evaluation, pre‑implementation research, study design

Translational success depends on intentional design work that makes key assumptions visible and testable before large investments are made. The Journal of Clinical and Translational Science (JCTS) is creating a dedicated home for this work through a new article type, *Translational Design Studies* [1].

Clinical and translational science has rightly invested in sophisticated trial designs, implementation frameworks, informatics, and engagement strategies. Yet many studies still struggle with recruitment, relevance, generalizability, and sustained uptake. We posit this is not because teams fail to execute, but because foundational questions and practical considerations about design were never answered sufficiently empirically to effectively shape what was built and how it was tested. Effective design must go beyond the art of study-by-study protocol development; we need an evidence-based framework for accelerating translation.

This editorial introduces and champions Translational Design Studies as rigorous scholarship that strengthens the entire translational pathway. These studies make design-phase evidence cumulative across settings and populations so that decisions about engagement and codesign, metrics, endpoints, eligibility, and delivery strategies are informed by data rather than by convention or optimism, while also attending to system- and policy-level features that may aid or impede implementation and scaling.

Design work is already happening across the translational continuum. Investigators and partners conduct qualitative inquiry with patients and clinicians, map workflows, assess feasibility and operational fit, validate candidate outcomes, examine readiness for change, and refine interventions through co-creation. Too often, however, these activities are based on historic patterns, and are fragmented, underreported, relegated to supplements, or forced into ill-fitting publication categories that obscure their scientific purpose.

The consequences are not abstract. When design uncertainties are not studied directly, trials may launch with unrealistic recruitment assumptions, measures that do not travel across contexts, interventions that collide with real-world constraints, or implementation plans that underestimate variation in systems and stakeholder priorities. Lost time and resources are only part of the cost; the field also risks damaging trust with those who did not feel engaged and loses the opportunity to learn systematically from why some designs flourish while others stall.

Translational Design Studies are intended to close this publishing gap by treating design as foundational science. In keeping with JCTS’s mission to advance the science of translation, this article type elevates empirical evidence about the choices that determine whether downstream translation can succeed.

A Translational Design Study can be defined simply: it is systematic inquiry conducted before, or alongside, planned translational work, with the explicit goal of generating generalizable empirical evidence that shapes what should be done, how it should be done, and with and for whom. Methodologically, these studies may be qualitative, quantitative, or mixed; what distinguishes them is their intent to inform consequential design decisions and reduce downstream risk.

Just as importantly, Translational Design Studies are not merely protocols that describe what a team plans to do without presenting the findings of design-relevant inquiry. They are not routine pilot studies limited to a single project’s feasibility, and they are not incomplete or negative efficacy studies repackaged after the fact. Rather, they follow scientific norms to make design choices, frequently grounded in implementation science (IS) principles and guided by a conceptual framework. Translational Design Studies seek to understand directional relationships between key constructs in the design so they can inform research strategies and support the testing of underlying assumptions. Translational Design Studies test assumptions, characterize context, and produce transferable insights about design choices.

Because these studies aim to advance the science of translation, rigor, and transparency are essential. Strong submissions will articulate a clear design question, specify what uncertainty or assumption is being tested, use reproducible methods, and present results that others can apply, adapt, and build on, even when the immediate motivation is a specific future trial or implementation effort.

Evaluation and continuous quality improvement (CQI) offer complementary perspectives that clarify why design matters. From an evaluation perspective, the logic model or theory of change is not a rhetorical accessory; it is a set of claims about how activities are expected to produce outcomes, under which conditions, and through which mechanisms [2]. Translational Design Studies provide a venue to test and refine those claims before definitive trials, making the pathway from inputs to impact more explicit and evidence-based.

CQI also reframes “iteration” as a mark of scientific maturity rather than a sign of failure. Approaches such as Plan–Do–Study–Act (PDSA) cycles formalize learning through small tests of change, measurement over time, and purposeful adaptation. Notably, the improvement literature also cautions that PDSA is often used without adequate documentation or adherence to its key principles, underscoring the need for rigorous reporting standards and clearer expectations [3].

A Translational Design Study can therefore be framed as a “prove to improve” contribution: it generates evidence to improve the design itself, not to provide a premature verdict on whether an intervention works. Such manuscripts can show, transparently, how early partner engagement and feedback, feasibility data, workflow constraints, or assessments of measurement validity or intervention fidelity lead to concrete refinements in the research approach, delivery strategy, implementation plan, or evaluation approach.

Statistical and analytic considerations are equally central to the credibility and usefulness of design-phase evidence. Translational Design Studies should test and describe methods of engagement, distinguish exploratory from confirmatory aims, specify the target estimand (population, intervention or exposure, comparator, outcome, and summary measure), and emphasize uncertainty quantification over ritualized hypothesis testing when the purpose is to inform design decisions.

Many translational designs now incorporate predictive or decision-rule components, including risk stratification, adaptive eligibility criteria, or tailored intervention delivery. When such components are proposed, Translational Design Studies can evaluate candidate models and decision rules using appropriate performance metrics (for example, discrimination and calibration), and apply internal validation strategies such as cross-validation or bootstrapping to reduce overfitting, with external validation when feasible to support generalizability. Transparent reporting standards, such as TRIPOD for prediction model studies and STROBE for observational designs, can improve interpretability and reuse of findings across settings [4, 5].

Simulation can also be a design-phase workhorse, allowing teams to explore how plausible effect sizes, variability, missing data, or operational constraints influence power, precision, or decision thresholds. Likewise, pre-specifying missing-data strategies, confounding control where relevant, and sensitivity analyses helps ensure that design recommendations rest on robust evidence rather than fragile analytic choices. The key expectation is linkage: analytic work should clearly inform downstream decisions about endpoints, eligibility, sample size justification, intervention tailoring, and the limits of generalizability.

Individual, family, and community engagement further strengthens the case for a design-focused publication home. The extensive work on community engagement has clarified that authentic, meaningful engagement with people and communities is central to trust, participation, and acceptance of results [6]. Expectations for meaningful engagement are rising across the research enterprise, and recent NIH, PCORI, and CDC efforts, including a roadmap for public engagement, the NExTRAC ENGAGE Working Group report, and the PCORI Foundational Expectations emphasize incorporating participant and public voices throughout the clinical research lifecycle. These developments make clear that engagement is not a single method to be included as a footnote, but a set of design decisions that must be adapted to condition, context, community priorities, and institutional infrastructure [7, 8].

Here, Translational Design Studies can advance a science of engagement by empirically testing how broad frameworks and principles translate into specific settings. Such studies might examine governance structures for engagement, mechanisms for compensating and supporting partners, measures of participation quality, or institution-level coordination that reduces burden on communities and enables alignment with community priorities. The PCORI Engagement Rubric offers one well-described framework for operationalizing engagement across research phases, and Translational Design Studies can extend this work by evaluating engagement strategies and metrics in diverse translational contexts [9]. The CDC/NIH Principles of Community Engagement offer a set of guiding principles that can be used to guide design decisions in ways that are valued by people across a wide range of communities, and build trust in the work done together [10].

The value proposition of Translational Design Studies is therefore broad and practical. Investigators gain a credible, peer-reviewed pathway to disseminate high-effort design work that often determines whether later studies can succeed. People and communities benefit when interventions and measures are shaped by lived priorities and context rather than retrofitted after a trial struggles. Health systems, funders, and the public benefit when design decisions are de-risked and strengthened early, reducing avoidable waste and increasing the probability that research produces usable, equitable impact.

For JCTS, this article type aligns directly with the journal’s role as a venue for advancing the science behind how translation happens, across the T0 to T4 continuum. Building a cumulative evidence base about design choices strengthens trials and implementation, and it also helps the field move beyond isolated success stories toward reproducible translational impact across settings and populations [11].

What should authors aim to include? A strong Translational Design Study will make the design decision explicit, describe the assumptions or uncertainties being tested, and present methods and evidence that readers can evaluate and reuse. It will connect findings to future or ongoing translational work, while also explaining what is transferable beyond the originating project, including what contextual features may moderate applicability. Where relevant, authors should consider using visual summaries, such as logic models, workflow schematics, decision-rule diagrams, or design-space maps, while keeping the core contribution anchored in reproducible evidence and interpretable implications.

This new article type also carries an invitation to reviewers and readers. Translational Design Studies should be evaluated primarily on the clarity and importance of the design question, the rigor and transparency of the methods, and the contribution to generalizable design knowledge, not on proximity to conventional endpoints. Read in that spirit, these studies become part of the infrastructure of translation, the evidence base that helps future teams design more feasible, relevant, and equitable research from the outset.

The work of translation does not begin at trial launch, nor at the first implementation milestone. It begins earlier, in the decisions about which groups to engage, how to engage them, what to build, how to build it, how to measure it, and how dissemination might best work. By providing a dedicated home for Translational Design Studies, JCTS aims to recognize, elevate, and accelerate the design-phase science that underpins high-impact translational research, and we invite investigators, methodologists, evaluators, and community partners to help build this cumulative science through submission in this new category.
